# The heterogeneous world of congruency sequence effects: an update

**DOI:** 10.3389/fpsyg.2014.01001

**Published:** 2014-09-09

**Authors:** Wout Duthoo, Elger L. Abrahamse, Senne Braem, Carsten N. Boehler, Wim Notebaert

**Affiliations:** ^1^Department of Experimental Psychology, Ghent UniversityGhent, Belgium; ^2^Department of Experimental Clinical and Health Psychology, Ghent UniversityGhent, Belgium

**Keywords:** cognitive control, congruency sequence effect, contingency learning, feature integration, conflict adaptation, repetition expectancy

## Abstract

Congruency sequence effects (CSEs) refer to the observation that congruency effects in conflict tasks are typically smaller following incongruent compared to following congruent trials. This measure has long been thought to provide a unique window into top-down attentional adjustments and their underlying brain mechanisms. According to the renowned conflict monitoring theory, CSEs reflect enhanced selective attention following conflict detection. Still, alternative accounts suggested that bottom-up associative learning suffices to explain the pattern of reaction times and error rates. A couple of years ago, a review by Egner (2007) pitted these two rivalry accounts against each other, concluding that both *conflict adaptation* and *feature integration* contribute to the CSE. Since then, a wealth of studies has further debated this issue, and two additional accounts have been proposed, offering intriguing alternative explanations. *Contingency learning* accounts put forward that predictive relationships between stimuli and responses drive the CSE, whereas the *repetition expectancy* hypothesis suggests that top-down, expectancy-driven control adjustments affect the CSE. In the present paper, we build further on the previous review (Egner, 2007) by summarizing and integrating recent behavioral and neurophysiological studies on the CSE. In doing so, we evaluate the relative contribution and theoretical value of the different attentional and memory-based accounts. Moreover, we review how all of these influences can be experimentally isolated, and discuss designs and procedures that can critically judge between them.

## INTRODUCTION

Over the last decades, the study of cognitive control – the flexible and adaptive regulation of our behavior – has increasingly drawn the attention of psychologists and neuroscientists alike. One critical aspect of this ability concerns the continuous monitoring of our behavior for situations in which missteps become more likely, allowing us to adjust behavior and prevent (further) deviation from goal-directed performance (i.e., conflict adaptation). The seminal *congruency sequence effect* (CSE) is considered a hallmark phenomenon of such cognitive control (Botvinick et al., 2001; see also Verguts and Notebaert, 2008, 2009). However, despite its central position in this research domain, the interpretation of the CSE is far from unequivocal, and alternative accounts highlighted the role of episodic memory (Hommel et al., 2004; Schmidt, 2013) or subjects’ explicit expectations (Gratton et al., 1992). Given the wealth of behavioral and neuroscientific studies relying on the CSE to further our insight in cognitive control, both in basic research and in more applied and clinical contexts, it seems of cardinal importance to recognize and dissociate these alternative views. Here, we give an overview of the studies that tested these accounts before we provide guidelines for further research.

The studies reviewed in the present paper investigated the CSE in typical conflict tasks such as the Stroop (Stroop, 1935), Eriksen flanker (Eriksen and Eriksen, 1974), and Simon (Simon, 1969) task. In these tasks, participants are asked to respond to a relevant stimulus feature (e.g., color), and the congruency between an irrelevant stimulus feature and either this relevant stimulus feature or the response is varied. The extent to which the irrelevant dimension is able to capture attention and influence performance is reflected in the size of the *congruency effect* – the difference between incongruent (I) and congruent (C) trials. This difference is typically strongly reduced when the previous trial was incongruent compared to when it was congruent – the CSE. In this review, we first elaborate on the standard interpretation of this CSE in terms of conflict adaptation and its underlying neural signature. Building further on a previous review by Egner (2007), we then set out to evaluate three alternative hypotheses for the conflict-monitoring theory: feature integration, contingency learning, and repetition expectancy. For each of these accounts, we highlight behavioral and neurophysiological evidence and discuss experimental procedures that can critically isolate their influence on the CSE. In the final section, we summarize the relative contribution of conflict adaptation, feature integration, contingency learning, and expectancy, and put forward some outstanding questions for further research.

## CONFLICT ADAPTATION

The CSE has been a major inspiration to the conflict-monitoring theory of Botvinick et al. (2001), which boosted and dominated research in the field of cognitive control over the last decade. Within this framework, it is assumed that fluctuations in the size of the congruency effect provide a direct window into online adjustments in cognitive control. The theory posits that the information processing stream is continuously monitored for the occurrence of conflict. Contingent upon the detection of conflict by the monitoring system, control is up-regulated. Following low conflict on congruent trials, control is temporarily down-regulated, and stronger interference effects on subsequent trials are predicted.

The CSE has proven to be a very robust and generalizable effect. Following its initial report in the context of an Eriksen flanker task (Gratton et al., 1992), it was replicated in a wide variety of tasks, including the color–word (e.g., Kerns et al., 2004), numerical (e.g., Cohen Kadosh et al., 2011), and gender face-word Stroop (e.g., Egner et al., 2008), the social (e.g., Kunde et al., 2012) and spatial Simon (e.g., Stürmer et al., 2002), the parity judgment (e.g., the spatial–numerical association of response codes or SNARC effect; Pfister et al., 2013), the picture–word interference (e.g., Duthoo et al., in revision), the perceptual fluency (e.g., Dreisbach and Fischer, 2011), the prime-target (e.g., Kunde and Wühr, 2006), and affective priming task (e.g., Frings and Wentura, 2008). Also in studies on arithmetics, difficulty arising from inappropriate strategy execution is susceptible to sequential, trial-to-trial performance adjustments (e.g., Uittenhove and Lemaire, 2012; Lemaire and Hinault, 2013). Notwithstanding the diversity of these experimental paradigms, the sequential effects are typically interpreted in terms of increased cognitive control following the detection of conflict.

The conflict-monitoring theory’s broad appeal can partly be attributed to the clear predictions it makes concerning the underlying brain mechanism involved in different cognitive control operations. According to Botvinick et al. (2001), the anterior cingulate cortex (ACC) is specifically involved in the detection of conflict (Jones et al., 2002), whereas subsequent control adjustments are implemented by the dorsolateral prefrontal cortex (DLPFC; Egner and Hirsch, 2005a). The CSE lends itself well to tease apart these brain regions, by comparing the neural response to incongruent trials dependent on whether the preceding trial was congruent or incongruent: the former trial transitions are supposed to evoke a strong conflict detection signal, whereas the latter trial transitions are associated with strong conflict resolution. fMRI investigations of the CSE in both the Stroop (Kerns et al., 2004) and Simon task (Kerns, 2006) convincingly showed that conflict-evoked ACC activity predicted subsequent behavioral adaptations, which were, in turn, accompanied by stronger DLPFC activity. In a follow-up study, Egner and Hirsch (2005b) elegantly showed that these behavioral adjustments are presumably brought about through cortical amplification of task-relevant information.

Other neurophysiological studies generally confirm the predictions of the conflict-monitoring theory. A series of EEG studies has uncovered deflections in event-related potentials that readily map onto the behavioral pattern of the CSE (for a recent review, see Larson et al., 2014). In a flanker task, sequential modulations of the ACC-mediated N2 component have been shown to covary with conflict adaptation effects in reaction times and error rates (Clayson and Larson, 2011, 2012; Forster et al., 2011; Larson et al., 2012). Similarly, the conflict slow potential elicited by incongruent Stroop trials is strongly reduced if the previous trial was incongruent compared to when it was congruent (Larson et al., 2009; Donohue et al., 2012). In the Simon task, Stürmer et al. (2002) showed smaller lateralized readiness potentials (LRPs) over the motor cortex following incongruent trials, indicating a reduced impact of the irrelevant dimension on response execution. In a follow-up repetitive transcranial magnetic stimulation (rTMS) study, Stürmer et al. (2007) demonstrated that the CSE was effectively abolished following TMS stimulation over the left DLPFC. Finally, Sheth et al. (2012) combined fMRI and human single neuron recording to show that modulation of the dorsal ACC by the previous trial predicts behavioral adaptation (i.e., a CSE). Moreover, these conflict adjustments were completely abolished following surgically targeted ablation of the dACC.

## FEATURE INTEGRATION

Despite being the dominant interpretation of the CSE, the conflict monitoring hypothesis has been challenged by alternative accounts in terms of episodic memory effects deriving from stimulus-response events, excluding a role for higher-level cognitive control processes. In essence, the feature integration account argues that the pattern of sequential modulation is problematically confounded with low-level repetition effects. Mayr et al. (2003), for example, pointed out that in a standard two-value arrow flanker task, exact stimulus repetitions will evoke priming effects that mimic the CSE. When they excluded these stimulus repetitions from the analyses, the CSE vanished. Hommel et al. (2004) took this idea one step further, by showing that not only complete, but also partial stimulus feature repetitions influence performance, again mimicking a CSE. Briefly, the feature integration account assumes that stimulus and response features of a current trial will be temporarily bound together into a common episodic memory representation. Activation of any of these features on the next trial will automatically co-activate the remaining features. Therefore, complete stimulus repetitions and complete stimulus alternations evoke faster responses, since no previous feature binding has to be undone. Critically, in a typical Simon or flanker task, comprising of four unique stimuli, sequential congruency, and feature integration are perfectly confounded: CC and II trial transitions entail complete stimulus repetitions or alternations, whereas CI and IC trial transitions always consist of partial stimulus repetitions.

In the wake of the feature integration account, extensive research efforts were dedicated to unraveling the relative contribution of higher-level attentional control and lower-level episodic memory effects to the CSE. A widely applied approach was to simply expand the stimulus set of a given congruency task and restrict the analysis to a subset of trials in which feature overlap was absent or kept equal. Still, studies that followed this logic drew some remarkably inconsistent conclusions. Even though the CSE was found to be completely abolished following *post hoc* exclusion of feature repetitions in some studies (Chen and Melara, 2009; Experiment 1 of Mayr et al., 2003; Nieuwenhuis et al., 2006; Fernandez-Duque and Knight, 2008), other studies reported a remaining CSE for transitions with equal feature overlap (Wühr, 2005) or devoid of any feature overlap (Kerns et al., 2004; Ullsperger et al., 2005; Kunde and Wühr, 2006). Notebaert and Verguts (2006), Akçay and Hazeltine (2007, 2011) and Bugg (2008) further removed negative priming trial transitions from the analysis (e.g., sequences where the irrelevant, to-be-ignored stimulus information of the previous trial becomes the relevant stimulus information on the next), and confirmed a contribution of attentional control to the CSE. Still, this experimental strategy is somewhat problematic: by excluding more and more trial transitions, the decision on the presence or absence of a CSE is made on an increasingly small and thus special subset of the data. In an attempt to circumvent this problem, Notebaert and Verguts (2007) proposed a multiple regression approach to statistically separate the influence of bottom-up feature repetitions and top-down control (see also Braem et al., 2012; Kunde et al., 2012). Another solution is to preclude trial transitions that are contaminated with feature integration *a priori*. Duthoo and Notebaert (2012) devised such eight-color vocal Stroop task devoid of any feature overlap and still found evidence for the CSE. Puccioni and Vallesi (2012; Experiment 1) ran a similar manual four-choice Stroop task. Again, a remaining CSE was found, yet only in the accuracy data.

However, both accounts are not mutually exclusive: The fact that a CSE is still found in the absence of feature repetitions does not imply that the feature integration account should be discarded (cf., Egner, 2007). Notebaert et al. (2006) elegantly demonstrated the additive contribution of both sources in a three-color manual Stroop task. By varying the response-to-stimulus interval (RSI), these authors were also able to show that bottom-up priming effects are evident at very short RSIs (i.e., 50 ms), whereas top-down, conflict-induced processes required more time to influence behavior (i.e., 200 ms). Given these insights, one can, however, wonder whether the neurophysiological evidence reviewed above is able to separate both contributions. Even though none of these studies were set up to test the predictions of the feature integration account, they did control for such effects in the analyses. Yet, as discussed by Egner (2007), predictions of the feature integration and conflict adaptation account crucially differ with respect to II sequences: whereas feature integration would predict these transitions to be associated with facilitation, being complete repetitions or alternations, conflict adaptation links these transitions with enhanced conflict resolution and controlled processing. The strong DLPFC activation in response to such transitions clearly favors the conflict adaptation hypothesis. Moreover, feature integration has no straightforward explanation as to why the CSE completely vanishes following TMS over the DLPFC (Stürmer et al., 2007) or surgical removal of the dACC (Sheth et al., 2012). As such, the feature integration account does not easily accommodate the interactions between ACC and DLPFC that lie at the core of the conflict-monitoring theory.

## CONTINGENCY LEARNING

Even though controlling for feature integration effects (be it *post hoc* or *a priori*) has become common practice in experiments on the CSE, this design choice actually comes at a price. Since most researchers decide to expand the stimulus set of their conflict tasks while at the same time maintaining a 50% congruent/incongruent ratio, they artificially increase the amount of congruent trials that would result from a random feature selection. Congruent trials would indeed occur less often, if stimulus features are selected randomly (e.g., 25% in a four-choice congruency task). As Mordkoff (2012) has argued, increasing the proportion of congruent trials forces irrelevant stimulus dimensions to become informative. In a Stroop task, for example, each (irrelevant) color word would then be more often paired with its congruent color than with any of the other colors. This association between a stimulus dimension and response is termed a contingency. Over time, such contingencies will render the stimulus dimension increasingly predictive of the correct response. Increasing the amount of congruent trials in a Stroop task would, for example, strengthen the association between the word “RED” and the corresponding response “red.” It has already been shown that participants are able to pick up and exploit such contingencies (see e.g., Dishon-Berkovits and Algom, 2000; Melara and Algom, 2003). This idea was elaborated upon in the work of Schmidt and Besner (2008) and Schmidt (2013), who claimed that contingency biases can artificially elevate the size of the CSE. More specifically, Schmidt et al. (2007) showed that high-contingency trials (i.e., predictive of the correct response) are responded to more rapidly and accurately than low-contingency trials, and that the difference between the two (i.e., the contingency effect) is larger following high-contingency trials compared to following low-contingency trials. In contingency-biased congruency tasks, congruent trials are high-contingency, so that the congruency sequence effect is perfectly confounded with the contingency sequence effect.

To illustrate the impact of these confounding contingency biases, Mordkoff (2012) compared performance on a contingency-unbiased (i.e., 25% congruent trials) and a contingency-biased (i.e., 50% congruent trials) four-choice Simon task. After removing all trial transitions involving feature repetitions, only the contingency-biased Simon task revealed a clear pattern of sequential modulation. Strikingly, there was no sign of a CSE in the contingency-unbiased task. In similar vein, Schmidt and De Houwer (2011) observed no remaining CSE in a Stroop task where all contingencies were kept equal. These observations led Schmidt (2013) to claim that conflict adaptation may simply be an illusion, and that the brain-behavior correlations that have been interpreted in support of the conflict-monitoring theory actually reflect the memory biases that alternative theories have put forward. ACC activity, for example, might then reflect contingency learning rather than conflict detection. Alternatively, Grinband et al. (2011) argued that the ACC is sensitive to time-on-task, irrespective of conflict. As such, every effect present in RTs (including the CSE) will correlate with ACC activity (cf., Schmidt, 2013). Even though there is no simple way of judging between these competing views on the basis of existing neurophysiological evidence, the lack of a behavioral effect in contingency-unbiased tasks poses a considerable challenge for a conflict adaptation account of the CSE.

As a critical test for conflict adaptation, Duthoo et al. (in revision) constructed versions of three common conflict tasks that controlled for both feature integration and contingency confounds *a priori*. To this end, a vocal six-color Stroop task was designed in which color and word never repeated across two consecutive trials and each word was equally often paired with its congruent color as with one of the five remaining incongruent colors. In this way, color–word contingencies were equated between congruent and incongruent trials, while the ratio of congruent/incongruent trials was kept at 50%. In similar vein, a six-letter manual flanker task was constructed. Finally, as to further minimize the contribution of memory biases, a picture–word interference task with 120 unique congruent and incongruent picture–word combinations was administered. Interestingly, a robust CSE was found in all three paradigms, notwithstanding the differences in response modality and conflict type. Moreover, this result was recently replicated in a similarly optimized four-choice flanker task (Hengstler et al., 2014). In order to account for the discrepancy with the findings of Schmidt and De Houwer (2011) and Mordkoff (2012), Duthoo et al. (in revision) point out that the introduction of a proportion congruent manipulation in contingency-unbiased designs (i.e., 75% incongruent trials) might have induced a sustained control state that potentially obscured the more transient control adjustments reflected in the CSE. Second, they argued that precluding memory biases by design might be crucial to observe “pure” cognitive control effects. This relates to the idea that conflict adaptation might be seen as a “last resort” which participants fall back on when simply relying on the environment (e.g., stimulus-response associations) is insufficient (Bugg, 2014).

Corroborating evidence comes from other recent studies showing significant sequential modulation in the absence of both stimulus/response repetitions and contingency learning. Kim and Cho (2014) let participants alternate between two color-flanker tasks. In one task, participants responded to vertically aligned red or yellow circles with two fingers, whereas in the other task they responded to horizontally aligned blue or green circles with two different fingers. As such, each trial transition never involved a response or stimulus repetition, and random selection of stimulus features produced a 50% congruent/incongruent ratio. When both response sets were assigned to one single hand (leading participants to process the two tasks as a single response mode), a significant CSE was obtained. In a similar vein, Schmidt and Weissman (2014) created a prime-target paradigm in which horizontally aligned stimuli (“<” or “>”) alternated with vertically aligned stimuli (“^∧^” or “v”). To rule out contingency confounds, four unique incongruent stimulus-distractor pairings were selected (i.e., “< >,” “> <,” “^∧^ v,” and “v ^∧^”). Even though the task comprised of different stimulus sets (horizontal vs. vertical) and response sets (left vs. right hand), Schmidt and Weissman (2014) found sequential modulation. Moreover, they replicated these findings in an identical task in which arrows were replaced with words (e.g., “up” or “left”). Weissman et al. (2014) found very similar results in an online replication study, as well as in an analogous contingency-unbiased Simon and temporal flanker task.

Finally, Freitas and Clark (2014) similarly restricted their analysis to transitions involving a shift in the vertical/horizontal dimension of their newly designed “Stroop-trajectory” paradigm, thereby excluding stimulus and response repetitions without introducing contingency confounds. On each trial, a series of identical, slightly overlapping pointing black triangles were presented one at the time in fast succession. Lastly, a smaller gray triangle pointing in the same direction was presented at either the top or bottom of the vertically aligned arrays, or at the left or right of the horizontally aligned arrays. Participants were asked to indicate the location of the smaller gray triangle, which either matched (congruent trials) or mismatched (incongruent trials) the direction in which the triangles were pointing. According to the authors, the gradual trial build-up in the task discouraged both negative priming and feature integration effects. Again, the authors reported strong sequential modulation. Taken together, the designs of Duthoo et al. (in revision), Freitas and Clark (2014), Kim and Cho (2014), Schmidt and Weissman (2014) and Weissman et al. (2014) suggest that a robust CSE can still be found, even when all known memory and learning confounds have been controlled for.

## REPETITION EXPECTANCY

In his review, Egner (2007) pointed out that the role of participants’ expectations has remained a strikingly underexplored factor potentially contributing to the CSE. Interestingly, in the original description of the CSE, Gratton et al. (1992) explained their findings in terms of strategic attentional adjustments driven by participants’ subjective expectations regarding the nature of the upcoming trial. Their *repetition expectancy* account assumed that participants were biased to expect repeating stimulus conditions over successive trials, regardless of the objective probability of these conditions to occur (Remington, 1969). Such repetition bias leads participants to expect that the trial following a congruent trial will be congruent, and the trial following an incongruent trial will be incongruent. Gratton et al. (1992) further theorized that such (passive) expectancies fed into (pro)active preparations that are not different from a situation in which congruency is explicitly cued: in anticipation of an incongruent trial, participants would focus their attention to the relevant dimension, whereas they would loosen their control settings in expectancy of a congruent trial. Such attentional filtering leads to fast responses to CC and II trial sequences, as expectancies are confirmed, but slow responses to CI and IC trials, since preparation misfires. The repetition expectancy account therefore predicts improved performance on congruency level repetitions, and impaired performance on congruency level alternations (i.e., a CSE). In contrast to the *reactive*, conflict-driven, more or less automatically induced control adjustments proposed by the conflict-monitoring theory, the repetition expectancy stresses the role of *proactive*, anticipatory, voluntary control processes.

Even though this theory quickly faded to the background of the theoretical discussion following the publication of the influential conflict-monitoring theory (Botvinick et al., 2001), a couple of studies has recently attempted to experimentally isolate the relative contribution of expectancy-induced controlled processes in a Stroop task, which yielded seemingly inconsistent findings. Duthoo and Notebaert (2012), for example, created experimental conditions that either favored or discouraged repetition expectancies (by raising the amount of congruency level repetitions or alternations, respectively) and looked for a transfer of these induced expectancies to a test phase in which congruency level repetitions and alternations were equally likely. The lack of a transfer effect suggested that participants failed to exploit the global transitional probabilities and prepare accordingly. Even in a context where congruency level alternations were highly probable, performance benefitted from scarce repetitions of congruent and incongruent trials. Jiménez and Méndez (2013) manipulated transitional probabilities in a similar way and also found that performance was not strongly affected by expectancies. As they also measured participants’ expectancies in separate blocks, the authors were able to show that even though expectancies aligned with the transitional manipulation, Stroop performance revealed a reaction time pattern in the opposite direction. Duthoo et al. (2013a), however, set out to test the prediction of the repetition expectancy account in a more direct fashion, by explicitly asking participants whether they expected an easy (congruent) or difficult (incongruent) trial before they responded to the Stroop stimulus. Over four experiments, results confirmed that participants displayed a repetition bias, expecting congruency level repetitions above chance level. Moreover, only when they predicted a congruency level repetition, a robust CSE was found. They concluded that expectancy can exert an influence on control above and beyond conflict-induced adjustments, yet only when these expectancies are explicitly manipulated or registered.

Another way to tease apart the relative contributions of reactive and proactive influences to the CSE is to examine their time course. Reactive, conflict-induced influences are assumed to be short-lived, transient and thus subject to decay over time, whereas proactive, anticipatory effects need some time for expectancies to build up and are therefore theorized to grow stronger or at least persist over time. By systematically varying the size of the RSI (between 500 and 5000 ms) and inter-stimulus-interval (ISI; between 500 and 7000 ms), Egner et al. (2010) demonstrated that CSEs are observed with small intervals (from 500 ms up to 2000 ms for RSI, and up to 3000 ms for ISI), yet completely disappear at the longer intervals. According to the authors, an interpretation in terms of conflict adaptation processes with a fairly steep decay function best fitted the data. Based on these data, van den Wildenberg et al. (2012) emphasized that adaptive cognitive control is inherently transient in nature. In a recent study, Duthoo et al. (2013b) replicated the reduced CSE with increasing intervals. However, they reasoned that expectancy-induced, proactive control is more likely to affect the CSE in situations that promote such control mode more strongly. To this end, they applied an RSI proportion manipulation that increased the probability of the stimulus appearing at the longer RSI. Under these conditions, they observed a reliable CSE for both short and long intervals, suggesting that proactive control can prevent the CSE from decaying rapidly.

Finally, a series of studies have manipulated expectancies more directly, by investigating the impact of explicit cues on the CSE. In their original paper reporting on the CSE, Gratton et al. (1992) already showed that a CSE-like pattern also emerged when applying probabilistic cues, and suggested that the previous trial’s congruency triggers a similar expectancy-driven attentional filtering mechanism as an explicit cue. This was later picked up by Aarts and Roelofs (2011) in an fMRI setting. They applied a similar probabilistic cueing procedure to a Stroop-like task to point out that anticipating upcoming conflict (or lack of conflict) can trigger similar sequential adjustments as experienced conflict (or lack thereof) on the previous trial. Interestingly, they not only replicated the CSE behaviorally, but also showed a similar sequential modulation of ACC activity that has been reported in previous fMRI studies on conflict-induced adjustments (Kerns et al., 2004; Kerns, 2006). The authors concluded that the ACC was involved in strategic allocation of cognitive control. An EEG study by Correa et al. (2009) also found that the ACC-mediated N2 deflection was reduced following cues that signaled high conflict. They theorized that anticipating conflict can speed up conflict detection and conflict resolution. Taken together, the neurophysiological data thus seem suggestive of a certain degree of neural overlap between the control networks triggered by reactive and proactive signals. However, such proactive control adjustments will be limited to these situations where expectancies are induced sufficiently strong or explicitly cued, suggesting that repetition expectancy cannot be the default interpretation of the CSE.

## CONCLUSION, GUIDELINES, AND OUTLOOK

Since its first report by Gratton et al. (1992), the CSE has boosted an extensive body of research that aimed to uncover the underlying mechanisms of sequential modulation, in order to better understand how people flexibly adapt their behavior. Based on the present literature review, some general conclusions can be drawn. First and foremost, consensus can be reached that both top-down, attentional adjustments and bottom-up, associative learning contribute to the (size of) the CSE. Moreover, their contributions seem to be largely dependent on the paradigm used to assess the CSE. In two-value congruency tasks, the relative share of feature integration will be substantial, if not complete (Mayr et al., 2003; Hommel et al., 2004; Bugg, 2008). When administering congruency tasks with more than two stimulus values, maintaining a 50% congruent/incongruent ratio introduces contingencies that will exert a strong influence on sequential effects (Schmidt and De Houwer, 2011; Mordkoff, 2012; Schmidt, 2013). Still, a series of recent studies (Duthoo et al., in revision; Freitas and Clark, 2014; Kim and Cho, 2014; Schmidt and Weissman, 2014; Weissman et al., 2014) has convincingly shown that in the absence of feature repetition and contingency learning confounds, a CSE can still emerge.

These studies allow distilling a set of guidelines on how to assess such relatively “pure” CSEs. First, the standard two-value congruency task has to be expanded to a four- (or more) value congruency task. Second, all transitions involving feature repetitions should preferably be excluded by design, rather than excluded *post hoc* or controlled for in the statistical analyses. Third, a 50% congruent/incongruent ratio should be installed while keeping all contingencies equal. One way of accomplishing this is to (a) create a unique set of incongruent stimuli (e.g., “RED” in green in a Stroop task, or “HHSHH” in a flanker task), so that irrelevant stimulus information is equally predictive of a congruent and incongruent response, and (b) constrain random selection of stimuli to avoid feature overlap (Duthoo et al., in revision; Hengstler et al., 2014). The major advantage here is that the classical conflict task remains intrinsically the same. Alternatively, four-value congruency tasks can be split up into a pair of two-value congruency tasks with separate stimulus-response mappings that alternate on a trial-by-trial basis (Schmidt and Weissman, 2014; Weissman et al., 2014). As such, stimulus features never repeat over successive intervals, and contingencies are kept equal across all trials. In a four-color Stroop task, for example, stimuli can be divided into two color pairs (and thus two sets of congruent and incongruent trials) that are presented in alternating fashion (e.g., Jiménez and Méndez, 2013; Weissman et al., 2014). Kim and Cho (2014) applied a similar strategy to a color-flanker task, but besides color and response they also varied the stimulus dimension (e.g., vertical vs. horizontal) on alternating trials. As a caveat, this manipulation only produced reliable CSEs when participants responded to all trials with one hand, and not when separate hands were used for horizontal and vertical trials. According to the authors, the latter led participants to no longer perceive the two tasks as involving a common “response mode.” It has indeed been well documented that increasing the difference between two tasks might hamper a transfer of control settings (i.e., a CSE) across tasks (for a review, see Braem et al., under revision).

Second, given that the evidence to date is indicative of a contribution of both attentional adjustments and episodic memory effects, the key theoretical question no longer pertains to which mechanism accounts for the CSE, but rather how these mechanisms interact and work together in producing adaptive behavior. Even if a CSE is still found in the absence of memory confounds, this does not at all imply that feature integration and/or contingency learning have no share in bringing about the CSE in designs that do not (fully) control for these. One promising avenue for further research therefore lies in parametrically manipulating these influences within the same experiment to systematically explore their contribution to the CSE. It could, for example, well be that inserting contingencies in the design precludes the need to engage in attentional control adjustments, as picking up and adapting to these regularities would be advantageous and less metabolically costly. In this light, Bugg (2014) has suggested that attentional control adjustments would constitute a “last resort” that participants will cling on when simpler learning mechanisms fail to produce satisfactory outcomes. To shed more light on this issue, one could also investigate whether participants will still pick up and rely on associative learning after they performed a congruency task in which such influences were controlled for, or vice versa (see Abrahamse et al., 2013, for an example of order effects on cognitive control strategies). The paradigms that were described above might serve as an excellent tool for such research endeavor.

Third, in almost all of the studies reviewed above, conflict-induced cognitive control has been the default explanation in situations where the CSE was found and confounding memory effects were controlled for. Rather than confirming the predictions of the conflict-monitoring theory, such findings mainly show that memory confounds cannot be the whole story. Therefore, the field is in need of observations that positively confirm specific predictions of the conflict-monitoring theory, and may at the same time benefit from a systematic exploration of other cognitive control processes that could (additionally) drive the CSE. In the present paper, we have evaluated one such additional source: repetition expectancy (Gratton et al., 1992). The studies reviewed above suggest that strategic, expectancy-based control adjustments only contribute to the CSE when they are induced sufficiently strong, and therefore cannot be the default explanation for the effect. Manipulating the proportion of congruency level transitions appeared too subtle (Duthoo and Notebaert, 2012; Jiménez and Méndez, 2013), whereas applying an RSI proportion manipulation (Duthoo et al., 2013a) and inserting probabilistic cues (Gratton et al., 1992; Aarts and Roelofs, 2011) or self-generated congruency predictions (Duthoo et al., 2013b) proved successful in eliciting strategic control adjustments. Such experimental manipulations provide an excellent research tool to investigate how proactive, expectancy-based control processes interact with reactive, conflict-induced control processes. A second additional source to the CSE that has recently gathered researchers’ attention is motivational in nature. More specifically, Botvinick (2007) hypothesized that the experience of cognitive conflict, or cognitive effort more generally, is inherently aversive, and that this negative value could modulate or even drive adaptations to conflict. A number of studies has indeed confirmed the first assumption, namely that that conflict is perceived as a negative event (Dreisbach and Fischer, 2012a; Lynn et al., 2012; Schouppe et al., 2012; Fritz and Dreisbach, 2013). However, whether it is this aversive nature (van Steenbergen et al., 2009), or rather the (conflict) resolution thereof (Braem et al., 2012; Schouppe et al., in press) that motivates adaptations to conflict, remains an open question (Dreisbach and Fischer, 2012b). There is substantial room for further research to investigate which of these components contribute to the CSE, and how they interact.

Fourth, once conflict adaptation has been clearly identified and demarcated, this would (re)activate some crucial challenges for further research. For example, it should be explored which precise mechanism(s) underlie such adaptation. These strategic adjustments could entail the altering of perceptual attention to target and/or distractor information (e.g., Egner and Hirsch, 2005b; Polk et al., 2008), the facilitation or inhibition of responses to target and/or distractor information (Ridderinkhof, 2002), or the general strengthening of active associations (i.e., Hebbian learning; Verguts and Notebaert, 2008, 2009). Furthermore, such strategic adjustments might also differ across (and depend on) specific tasks used to assess the CSE (Egner, 2008), and should ideally be thoroughly explored in unbiased designs (e.g., Weissman et al., 2014). Another challenge would be to investigate how domain-specific the mechanism(s) underlying conflict adaptation is/are. Indeed, earlier studies already explored this potential for transfer across different tasks, contexts and/or conflict types, spawning an interesting, yet seemingly inconsistent set of results (for reviews, see Braem et al., under revision; Egner, 2008). Although most studies appear to demonstrate domain-specific CSEs, others claim that adaptations to conflict can be domain-general. Therefore, it remains to be investigated which general principle(s) this transfer adheres to (Braem et al., under revision). To further test the generalizability of the conflict adaptation mechanisms, it should also be explored if adaptation by *recent* conflict (i.e., the CSE) relies on a similar mechanism as adaptation to *frequent* conflict, or not. The latter is often studied in proportion congruency tasks, where specific proportions of (in)congruent trials modulate the congruency effect – and thus presumably the amount of cognitive control. Interestingly, the literature on this proportion congruency effect sparked similar debates as for the CSE reviewed here. For example, it has been discussed if the proportion congruency effect reflects conflict adaptation or merely S-R contingency learning processes (Abrahamse et al., 2013; Schmidt, 2013; see Bugg and Crump, 2012 for a review). Similar to the CSE, the empirical evidence seems to suggest that contingency learning cannot account for all observations – and thus that conflict adaptation seems to be involved. However, it is still strongly debated if the CSE and the proportion congruency effect involve the same underlying mechanism (Verguts and Notebaert, 2008, 2009) or not (Funes et al., 2010a,b; Torres-Quesada et al., 2013; Wühr et al., in press). These types of questions should be (re)considered using the appropriate designs for assessing CSEs.

Finally, the neurophysiological data on the CSE to date do seem to nicely confirm predictions of the conflict monitoring account: Both imaging results (Kerns et al., 2004; Egner and Hirsch, 2005a,b; Kerns, 2006; but see Grinband et al., 2011), EEG data (Stürmer et al., 2002; Larson et al., 2009, 2012; Clayson and Larson, 2011, 2012; Forster et al., 2011; Donohue et al., 2012; but see Wendt et al., 2007), as well as an rTMS (Stürmer et al., 2007) and human single neuron recordings and lesion study (Sheth et al., 2012) are consistent with the proposed ACC-driven conflict detection and DLPFC-implemented conflict resolution control processes. However, all of these studies have employed congruency tasks that were critically contaminated with feature integration and/or contingency learning confounds. Even though no study has yet set out to directly test the neural correlates of these learning accounts, it may well be the case that medial and dorsolateral prefrontral cortex are also critically involved in such associative learning (Grandjean et al., 2013; Schmidt, 2013). The unbiased designs discussed above might therefore be an excellent starting point for further neurophysiological studies that want to elucidate the respective roles of the ACC and DLPFC in producing a CSE. A final challenge for future research then lies in integrating conflict-control and associative learning mechanisms, as well as their interactions and neural substrates into overarching models of cognitive control (see, e.g., Verguts and Notebaert, 2008, 2009).

## Conflict of Interest Statement

The authors declare that the research was conducted in the absence of any commercial or financial relationships that could be construed as a potential conflict of interest.
